# The chromosome‐scale high‐quality genome assembly of *Panax notoginseng* provides insight into dencichine biosynthesis

**DOI:** 10.1111/pbi.13558

**Published:** 2021-02-14

**Authors:** Zijiang Yang, Guanze Liu, Guanghui Zhang, Jing Yan, Yang Dong, Yingchun Lu, Wei Fan, Bing Hao, Yuan Lin, Ying Li, Xuejiao Li, Qingyan Tang, Guisheng Xiang, Simei He, Junwen Chen, Wei Chen, Zhongping Xu, Zichao Mao, Shengchang Duan, Shuangxia Jin, Shengchao Yang

**Affiliations:** ^1^ State Key Laboratory of Conservation and Utilization of Bio‐Resources in Yunnan The Key Laboratory of Medicinal Plant Biology of Yunnan Province, National and Local Joint Engineering Research Center on Germplasm Innovation and Utilization of Chinese Medicinal Materials in Southwest China Yunnan Agricultural University Kunming China; ^2^ Yunnan Plateau Characteristic Agriculture Industry Research Institute Kunming China; ^3^ National Key Laboratory of Crop Genetic Improvement Huazhong Agricultural University Wuhan China; ^4^ College of Agronomy and Biotechnology Yunnan Agricultural University Kunming China; ^5^ NOWBIO Technology Co. Ltd Kunming China

**Keywords:** *Panax notoginseng*, genome, triterpenoid saponins, dencichine

*Panax notoginseng* (2n = 2x = 24), reputed as a valuable medicinal plant, belongs to the Araliaceae family (Figure 1a). *P. notoginseng* has been used as a traditional Chinese medicine with obvious efficacy and favourable safety. Currently, *P. notoginseng* is an important material of many well‐known Chinese patent medicines including Xuesaitong, Yunnan Baiyao and Compound Danshen Dripping Pills (Xu *et al.,* 2019). Triterpenoid saponins (TSs) and dencichine are the main bioactive compounds in *P. notoginseng*. The therapeutic effects of TSs include anti‐inflammatory, vasorelaxation and anticancer. Dencichine is used for treatment of injury‐induced trauma, and its haemostatic function was proven by clinical practice (Ng, 2006).

**Figure 1:**
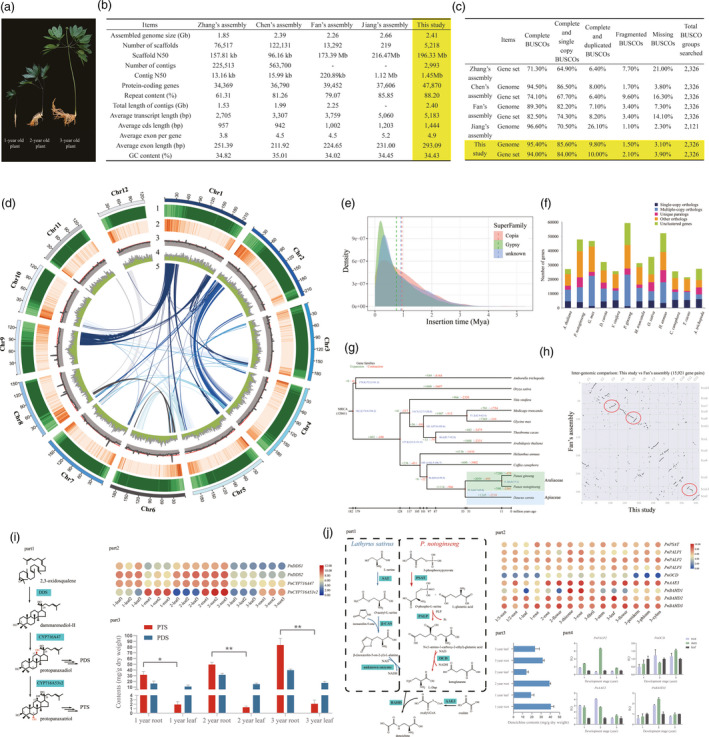
Analysis of *Panax notoginseng* genome. (a) *P*. *notoginseng*. (b) *P. notoginseng* assemblies comparison. (c) BUSCO results comparison. (d) Circular map of *P*. *notoginseng*. 1) Transposable elements, 2) gene density, 3) depth distribution of Illumina reads, red line indicate the expected depth, 4) GC content and 5) synteny relations. (e) Insertion times of LTR families. (f) Gene families analysis. (g) Phylogenetic analysis of *P*. *notoginseng* with estimated divergence time and gene families expansion / contraction. (h) Chromosome synteny of Fan’s assembly and this study. (i) Part 1 Biosynthesis pathway for TSs. Part 2 Expression heatmap of genes involved in TSs biosynthesis. The 1, 2 and 3 year indicate the age of *P. notoginseng* and the 1, 2 and 3 suffix indicate 3 biological replicates. Part 3 Contents of PDS and PTS in *P. notoginseng*. PDS including ginsenosides Rb1 and Rd; PTS including notoginsenoside R1 and ginsenosides Rg1 and Re. Error bars indicate standard deviation. * and ** indicate significant differences at *P* < 0.05 and *P* < 0.01. (j) Part 1 Biosynthesis pathway for dencichine. Part 2 Expression heatmap of genes involved in dencichine biosynthesis. Part 3 Contents of dencichine in *P. notoginseng*. Error bars indicate standard deviation. Part 4 Real‐time quantitative PCR of four genes involved in dencichine biosynthesis

Four versions of *P. notoginseng* genome assembly have been reported (Chen *et al.,* 2017; Fan *et al.,* 2020; Jiang *et al.,* 2020; Zhang *et al.,* 2017). The first two assemblies are highly fragmented, which limits genomic studies of *P. notoginseng*. The continuity was significantly improved in Fan’s and Jiang’s assembly (Figure 1b). The genome size of *P. notoginseng* has been estimated to be about 2.31 Gb using flow cytometry. While, the size of four assemblies varied between 1.85 to 2.66 Gb. Here, we present a chromosome‐scale assembly for *P. notoginseng* created using PacBio long reads, Illumina short reads and Hi‐C technology. The heterozygosity of *P. notoginseng* was estimated to be 0.23%, indicating its low genetic variability. The size and contig N50 are 2.41 Gb and 1.45 Mb, respectively (Figure 1b). Approximately 87% of the contigs were anchored into 12 pseudo‐chromosomes. The result showed that 2,219 out of 2,326 (95.4%) eudicots BUSCOs were found to be complete in the assembly with a decent redundancy (9.8% complete and duplicated BUSCOs) (Figure 1c). Our assembly is more complete compared with Fan’s assembly (89.3% complete BUSCOs). Since the data of Jiang’s assembly are not currently available (to be released in September, 2021), only genome BUSCO result with 2,121 BUSCOs data set was presented. The percentage of complete BUSCOs in Jiang’s assembly is 96.6%, yet the complete and duplicated BUSCOs are relatively high (26.1%) for a diploid species with low heterozygosity. Considering the assembled size of Jiang’s assembly is 15.2% percent larger than the estimated genome size, Jiang’s assembly might contain a large portion of differentiated haplotype sequences. We also aligned ~68X Illumina short reads and 266,984 RNA transcripts to our assembly and found 98.4% coverage rate and 96.0% mapping rate, respectively. Specifically, the depth distribution of mapped Illumina reads showed no anomaly (Figure 1d).

Annotation revealed 88.2% of the genome as repetitive elements. Transposable elements (TEs) were identified as the most abundant, spanning 87.8% of the assembly. Among the subfamilies of TEs, long terminal repeats (LTRs) took up 81.2% of the assembly. Insertion time analysis of 24,861 intact LTRs revealed *P. notoginseng* experienced a burst increase of LTRs started 1 million years ago (Mya) (Figure 1e). The LTR assembly index (LAI) reached 12.44, validating the high quality of our assembly. A total of 47,870 genes were predicted in *P. notoginseng*. Compared with previous studies, more genes were predicted in *P. notoginseng* (Figure 1b). Comparison of gene set BUSCO results showed our gene set as the most complete (94% complete BUSCOs) (Figure 1c), which might explain the higher number of predicted genes in our assembly. Among the predicted genes, 98.51% were supported by RNA‐seq data and 94.61% could be annotated in public database. The gene number was smaller than the closely related species *Panax ginseng* (59,352 genes), probably due to the tetraploid nature of *P. ginseng* (Kim *et al.,* 2018).

Gene family analysis of *P. notoginseng* and 11 other angiosperms suggest *P. notoginseng* genes were clustered into 17,306 families and *P. notoginseng* had much less multiple‐copy orthologs compared with *P. ginseng* (Figure 1f). Phylogenetic tree based on single‐copy genes suggest *Panax* genus diverged from the Apiaceae species *Daucus carota* 44.7–65.0 Mya, and divergence of *P. notoginseng* and *P. ginseng* is between 6.0 ‐17.1 Mya (Figure 1g). Chromosome synteny analysis of Fan’s assembly with ours showed many discontinuities and segmental inversions (Figure 1h), where most of these anomalies fell into TE‐rich regions (Figure 1d). This suggests limitations of current technologies in assembling highly repetitive plant genomes.

Three key enzyme families are involved in biosynthesis of TSs: oxidosqualene cyclases (OSCs), cytochrome P450 (CYPs) and glycoltransferases (GTs). Dammarenediol‐II synthase (DDS) from OSCs family catalyses the cyclization of 2,3‐oxidosqualene, forming the triterpene scaffolds dammarendiol‐II. Then, dammarendiol‐II was modified by CYPs and GTs through hydroxylation and glycosylation of certain positions (mainly C‐3, C‐6 and C‐20). Depending on whether C‐6 contains a hydroxyl group, TSs are divided into protopanaxadiol saponins (PDS) and protopanaxatriol saponins (PTS) (Figure 1i part1). Functional studies revealed that *CYP716A47* and *CYP716A53v2* are responsible for biosynthesis of PDS and PTS, respectively (Kim *et al.,* 2015). *DDS*, *CYP716A47* and *CYP716A53v2* were all identified in *P. notoginseng* genome. Specifically, *PnDDS1* and *PnDDS2* were derived from proximal duplication (separated by two genes on chromosome 3). Unlike *P. ginseng*, PTS are abundant in roots while scarce in leaves in *P. notoginseng* (Figure 1i, part 3). RNA expression of key genes was investigated to unveil the mechanism of tissue‐specific PTS distribution. No tissue‐specific expression patterns were found for *DDS* and *CYP716A47*, whereas the expression level of *CYP716A53v2* was significantly higher in roots than in leaves (Figure 1i part2). This suggests the differential expression of *CYP716A53v2* in leaves and roots is responsible for the imbalance PTS distribution.

Dencichine is a non‐protein amino acid present in *Panax*, *Lathyrus* and several other species. In *Lathyrus sativus*, the biosynthesis of dencichine involves L‐serine, which is transformed into *O*‐acetyl‐L‐serine via serine acetyltransferase (SAT). β‐cyanoalanine synthase (β‐CAS) catalyses the formation of β‐(isoxazolin‐5‐on‐2‐yl)‐L‐alanine (BIA) using *O*‐acetyl‐L‐serine and isoxazolin‐5‐one. BIA is proposed to be converted into L‐2,3‐Diaminopropionic acid (L‐Dap). Finally, dencichine is synthesized from L‐Dap by enzymes from BAHD acyltransferase family. (Figure 1j, part1) (Yan *et al.,* 2006). The intermediates isoxazolin‐5‐one and BIA were not detected in *P. notoginseng*, indicating the mechanism of dencichine biosynthesis might be different from *L. sativus*. In *Staphylococcus aureus*, two enzymes from Class II PLP‐dependent enzymes (PALP) and ornithine cyclodeaminase (OCD) family could produce L‐Dap using *O*‐phospho‐L‐serine (Kobylarz *et al.,* 2014). Based on these findings, we proposed a novel biosynthetic pathway for dencichine in *P. notoginseng* involving five different type of enzymes: 3‐phosphoserine aminotransferase (PSAT), PALP, OCD, acyl activating enzyme 3 (AAE3) and BAHD. Candidate genes for these five enzymes were identified in our study. Notably, dencichine is found to be more abundant in rhizome, fibril and root of *P. notoginseng* whereas less in leaves (Figure 1j, part3), which is in accordance with expression profiles of candidate genes (Figure 1j, part2). Additionally, we measured the expression levels of several candidate genes and found no tissue‐specific expression patterns using real‐time quantitative PCR (Figure 1j, part4).

In conclusion, this high‐quality genome assembly of *P. notoginseng* provides novel insights into unique saponins distribution pattern and reveals possible dencichine biosynthetic pathway.

## Author contributions

S. Y. and S. J. conceived the study. Z. Y., G. L., G. Z., J. Y., Y. D., Y. L., W. F., B. H., Y. L., Y. L., X. L., Q. T., G. X., S. H., J. C., W. C., Z. X., Z. M. and S. D. performed the experiments and carried out the analysis. Z. Y., G. L., G. Z., S. Y. and S. J. designed the experiments and wrote the manuscript. All the authors approved the manuscript.

## Conflict of interest

No conflict of interest declared.

## Data Availability

Raw sequencing data have been deposited in NCBI under BioProject number PRJNA608068. Genome assembly and annotation have been deposited in Herbal Medicine Omics Database (http://herbalplant.ynau.edu.cn/).
